# Lactoferrin as an antimicrobial against *Salmonella enterica* and *Escherichia coli* O157:H7 in raw milk

**DOI:** 10.3168/jdsc.2020-0030

**Published:** 2021-03-12

**Authors:** Erika N. Biernbaum, Anita Gnezda, Samina Akbar, Rose Franklin, Paul A. Venturelli, John L. McKillip

**Affiliations:** 1 Department of Biology, Ball State University, Muncie, IN 47306; 2 Department of Chemistry, Ball State University, Muncie, IN 47306; 3 Department of Biomedical Sciences, Marian University, Indianapolis, IN 46222

## Abstract

Improper storage conditions or processing of milk leads to potential spoilage and illness, due in part to temperature abuse, allowing bacteria present to grow and spoil the product. However, certain proteins naturally found in raw milk, such as lactoferrin, have reported antibacterial properties. The levels of lactoferrin required to effectively inhibit growth of pathogens have not been investigated thoroughly. This study aimed to examine various concentrations of lactoferrin as a potential biopreservative and as an antimicrobial against the common dairy pathogens *Salmonella enterica* and *Escherichia coli* O157:H7. Minimum inhibitory concentration assays were conducted on raw bovine milk in which the bacteria were exposed to varying concentrations of lactoferrin. In the raw milk system, the growth of *E. coli* O157:H7 was significantly decreased at levels greater than 14.05 mg/mL lactoferrin based on the reduction of tetrazolium salts. For *S. enterica*, only lactoferrin concentrations at or above 112.5 mg/mL in the milk resulted in reduced growth. Taken together, these results indicate that lactoferrin may have biopreservative potential. To fully examine the practicality and effectiveness of lactoferrin as an antimicrobial additive, a similar study should be conducted using additional (gram-positive) pathogens, such as *Bacillus cereus* and *Listeria monocytogenes*. If effective, lactoferrin could prolong the shelf life of dairy products and help reduce the incidence of foodborne illnesses in developing countries with limited refrigeration capability.

## Highlights

• In the absence of proper refrigeration of raw milk, bacterial pathogens such as *Salmonella* and *E. coli* O157:H7 may cause illness.
• Growth of each pathogen in raw milk was measured using a novel tetrazolium salt reduction assay.
• Growth of *Salmonella* and *E. coli* O157:H7 was inhibited by lactoferrin in raw bovine milk.
• Lactoferrin represents a safe means of milk preservation in developing countries lacking proper refrigeration.

## Graphical Abstract

**Figure:**
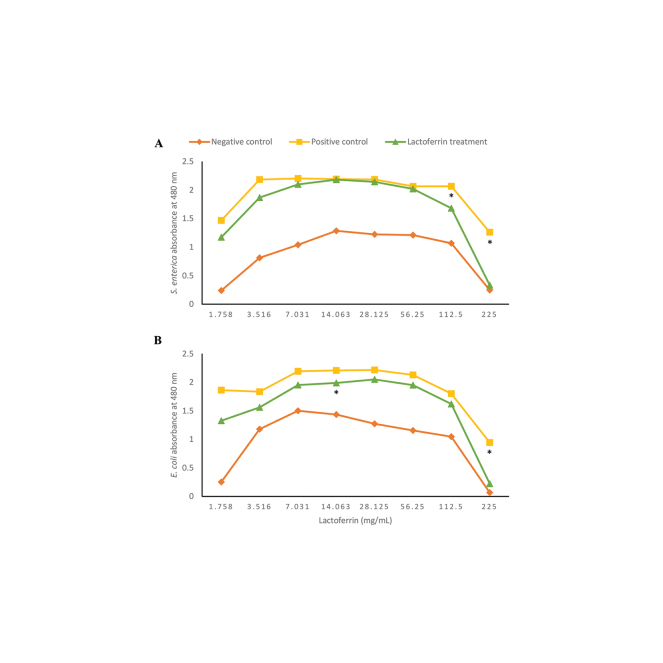
**Summary:** Natural antimicrobials can be added to help extend the shelf life of milk and reduce contamination potential, especially in developing countries. The levels of lactoferrin found in raw milk have not been thoroughly studied in regard to specific antibacterial activity against key pathogens. This study aimed to investigate various concentrations of lactoferrin as a biopreservative measure against *Salmonella enterica* and *Escherichia coli* O157:H7. Our results indicate that lactoferrin has potential as a biopreservative. These results will assist in the reduction of milk-borne illnesses in developing countries that may lack means for proper refrigeration.

Although the consumption and popularity of dairy milk may be declining in the United States in recent years, demand is slowly increasing in developing countries. Dairy milk has long been considered a staple in Western households, but it plays a much larger role in developing countries, such as India, China, and sub-Saharan Africa. Milk production is one way for families to increase their livelihoods while also providing a steady income. Consumption of dairy milk also helps combat malnutrition in developing countries by providing key nutrients such as calcium, magnesium, and vitamins. However, if dairy milk or milk products are not processed or handled properly, the likelihood of contamination increases. Common bacterial dairy pathogens include *Bacillus cereus, Listeria monocytogenes, Campylobacter jejuni, Escherichia coli*, and *Salmonella*. The Centers for Disease Control and Prevention estimates that 48 million people in the United States contract a foodborne illness each year.

*Salmonella enterica* is a routine foodborne pathogen, causing 93.8 million cases of salmonellosis annually worldwide. To aid in infection, *S. enterica* has a type III secretion system containing pathogenicity islands 1 and 2 to help with invasion and survival in host cells (Desin et al., 2009; Singh et al., 2018). *Salmonella enterica* also contains an *spv* operon that encodes genes functioning in translocation, actin polymerization, and host cell apoptosis (Guiney and Fierer, 2011; Derakhshandeh et al., 2013). A similar bacterium that can also cause foodborne illnesses is *E. coli* O157:H7 (enterohemorrhagic *E. coli*; **EHEC**). In the United States, EHEC causes an estimated 73,000 cases annually. Although not as common of a dairy pathogen as *Salmonella*, EHEC can cause more severe infections, such as hemolytic uremic syndrome, which affects the blood vessels and platelets of the host and can ultimately lead to anemia and kidney failure (Robinson and McKillip, 2010). The severity of an EHEC infection is due in part to its 3 main virulence determinants: pO157 plasmid, locus of enterocyte effacement, and *stx1/2* genes. These virulence factors aid EHEC in attaching to host epithelial cells, forming lesions in the intestines, and causing cytotoxicity in a variety of host cell types (Lim et al., 2010; Battle et al., 2014).

To help reduce the possibility of dairy milk becoming contaminated with such types of bacterial pathogens, natural antimicrobials could be added after processing as a biopreservative measure. One such protein with biopreservative potential is lactoferrin. Lactoferrin is a naturally occurring protein in mammalian excretory fluids, such as saliva, tears, and milk (Farnaud and Evans, 2003). The concentration of lactoferrin varies from mammalian species and type of body fluid, with bovine milk containing one of the lowest concentrations (0.2–0.4 mg/mL). This protein has various properties noted by several studies, including antiviral, antifungal, anti-inflammatory, and antimicrobial (Farnaud and Evans, 2003; Hao et al., 2018). Wang et al. (2019) reviewed the antimicrobial actions of lactoferrin, including the binding of the lipid A portion of the LPS layer of gram-negative bacteria, targeting teichoic acid in gram-positive bacteria, and sequestering iron to reduce the amount available for pathogens. Despite understanding lactoferrin's mechanistic actions, little research has been conducting regarding its ability to act as an antimicrobial in milk at the natural levels.

The goal of this study was to test the effectiveness and practicality of lactoferrin as an antimicrobial against 2 common dairy pathogens. We hypothesized that the natural levels of lactoferrin would inhibit the growth of both *S. enterica* and *E. coli* O157:H7. If proven to be effective, lactoferrin could be used as a biopreservative measure in developing countries, thus reducing the incidence of foodborne illnesses and dairy spoilage.

Cultures of *S. enterica* ssp. *enterica* serovar Enteritidis (ATCC 13076) and *E. coli* O157:H7 (ATCC 43895) were streaked on tryptic soy agar (Fisher Scientific) and incubated overnight at 37°C. One isolated colony from each plate was transferred to 5 mL of tryptic soy broth (**TSB**; Fisher Scientific) and incubated for 24 h at 37°C. This was repeated once more, and the optical density (A_550_) of the cultures was measured to determine the concentration (cfu/mL) correlated with standard plate count on each bacterial species using tryptic soy agar and incubation at 37°C.

A broth assay was performed to test the natural levels of bovine lactoferrin as an antimicrobial against *S. enterica* and *E. coli* O157:H7. The bacterial cultures were grown to a concentration of 10^2^ cfu/mL in 5 mL of TSB, serving as a positive control. The treatment consisted of cultures (10^2^ cfu/mL) exposed to 0.2 and 0.4 mg/mL lactoferrin. Samples were incubated on a shaker (150 rpm) at ambient temperature (23°C) and at 37°C. The optical density (A_600_) of each culture was measured every hour for 18 h, as was the survival of each bacterial species.

To determine the lactoferrin concentration that would inhibit the growth of *S. enterica* and *E. coli*, a MIC assay was performed. Each of the following replicates were done in triplicate in a transparent, flat-bottomed 96-well plate (Corning Inc.), with each well containing 50-µL total volume. Uninoculated TSB served as a negative control, inoculated TSB (10^2^ cfu/mL *S. enterica* or *E. coli*) served as a positive control, and the experimental sample consisted of inoculated TSB (10^2^ cfu/mL *S. enterica* or *E. coli*) and varying concentrations of bovine lactoferrin (0.1% saturated, Sigma-Aldrich L4765). The lactoferrin was serially diluted in a 1:2 fashion, beginning with 225 mg/mL and ending with 1.75 mg/mL. The plates were covered with parafilm and incubated at 37°C for 48 h. The absorbance was then measured at a single 48-h time point at 550 nm using a microtiter plate reader (Tecan).

Raw bovine milk was screened for naturally occurring *S. enterica* and *E. coli* O157:H7 24 h before the assay using the standard plate count method on *Salmonella* shigella agar (Fisher Scientific) and sorbitol MacConkey agar (Fisher Scientific). The raw milk was then heat treated to kill any microorganisms in the milk and to denature the native lactoferrin. The milk was treated in an 85°C water bath for 15 min, with swirling every 2 min, and incubated at room temperature (23°C) for 24 h to allow germination of bacterial spores if present in the milk. The milk was heat treated once more the following day for 15 min at 85°C to deactivate the spores and then transferred to the wells of a 96-well plate. Tetrazolium salts (0.05% wt/vol; 2,3,5-triphenyltetrazolium chloride, Sigma-Aldrich T8877) were added to the negative and positive controls and to the lactoferrin-treated wells for colorimetric purposes. The microtiter plate was set up in the same manner as the broth MIC assay as noted above. After 48 h of incubation, the absorbance (A_480_) of the wells was measured.

We used a mixed-effects model to analyze the effect of lactoferrin (treatment vs. positive control) on the absorbance of bacterial cultures for both MIC assays. Treatment was a fixed factor, and both microtiter plate number and row were mixed effects. We carried out the analysis via the lme4 package (Bates et al., 2015) in version 3.5.2 of R statistical software (R Core Team, 2018) and assessed statistical significance at α = 0.05.

The changes in absorbance for *S. enterica* and *E. coli* O157:H7 after 48 h of exposure to lactoferrin in TSB are shown in Figure 1. The inhibitory activity of lactoferrin was best observed against *E. coli* O157:H7, as the mean absorbance was consistently lower than that of the positive control, except for 1.76 and 225 mg/mL lactoferrin levels. The overall growth of *E. coli* O157:H7 decreased as the concentration of lactoferrin increased. The MIC of lactoferrin against *E. coli* O157:H7 was ≤3.516 mg/mL (*P* = 0.007). A similar trend was seen for *S. enterica*. The density of *S. enterica* was lower than the positive control and decreased with the addition of lactoferrin, except for 1.758 mg/mL. Based on statistical data, the MIC for lactoferrin increased against *S. enterica*, with 7.031 mg/mL (*P* = 0.04). It is noteworthy that levels of lactoferrin naturally found in the raw milk did not adversely affect growth rate or bacterial density for either pathogen (data not shown).

**Figure 1:**
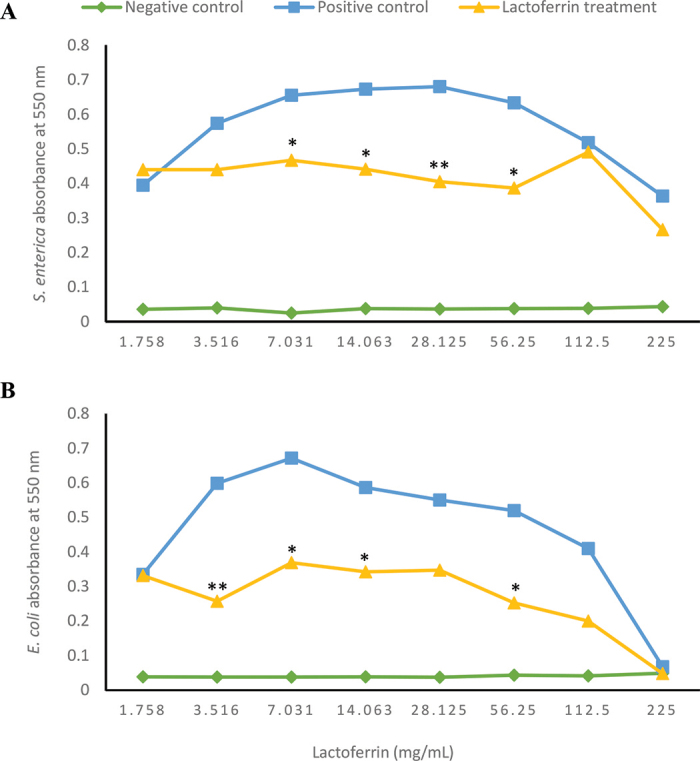
Antimicrobial activity of lactoferrin against *Salmonella enterica* (A) and *Escherichia coli* O157:H7 (B) in tryptic soy broth. The addition of lactoferrin, apart from 1.758 mg/mL, decreased the absorbance of both bacteria after 48 h compared with the positive control. The lactoferrin MIC was ≤7.031 mg/mL for *S. enterica* and ≤3.516 mg/mL for *E. coli* O157:H7. A significant absorbance difference compared with the positive control is indicated by * (*P* < 0.05) and ** (*P* ≤ 0.01).

The effect of lactoferrin on *S. enterica* and *E. coli* O157:H7 in milk is shown in Figure 2. The average absorbances using the colorimetric assay (reduction of tetrazolium salts) of both bacteria in bovine milk were more variable than what was observed in TSB. The average absorbances (tetrazolium salt reduction) of *S. enterica* treated with the middle concentrations of lactoferrin were extremely similar to the positive control values, which lacked lactoferrin. The 2 highest concentrations resulted in a decreased absorbance, resulting in an MIC of 112.5 mg/mL (*P* = 0.06). The absorbance readings also decreased for *E. coli* O157:H7 with the addition of lactoferrin. Each concentration tested inhibited the growth of *E. coli*, as seen by a slight decrease in absorbance compared with the positive control, although not all these values were statistically significant, only 225 and 14.063 mg/mL (*P* = 0.03).

**Figure 2:**
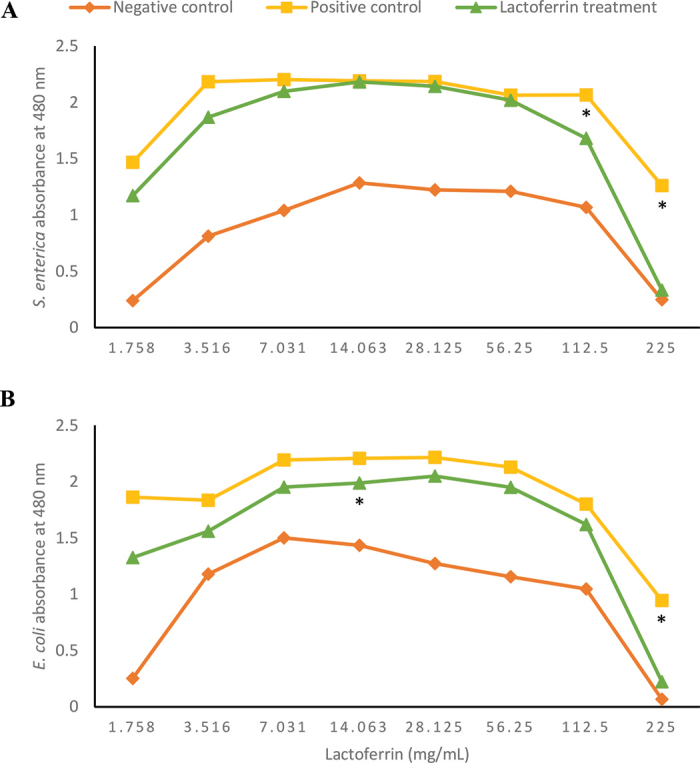
Absorbance of *Salmonella enterica* (A) and *Escherichia coli* O157:H7 (B) in bovine milk with varying concentrations of lactoferrin. The average density of *S. enterica* was more variable than that of *E. coli* O157:H7. The absorbance for the positive control and lactoferrin-treated *S. enterica* was very similar, with the 2 highest and lowest concentrations slightly inhibiting growth. The effect of lactoferrin worked best on *E. coli* O157:H7, as each concentration decreased the average absorbance. The MIC was 112.5 mg/mL for *S. enterica* and O157:H7 14.063 mg/mL for *E. coli*. A significant absorbance difference compared with the positive control is denoted by * (*P* ≤ 0.05).

We chose to study lactoferrin as a potential biopreservative because of its reported antimicrobial properties (Niaz et al., 2019). Lactoferrin is found in a variety of mammalian sources, including saliva, tears, milk, and other excretory fluids. The activity of lactoferrin as an antibacterial is partly due to its ability to sequester iron (Fe^3+^) away from bacteria. Bacteria use iron in DNA and RNA synthesis, the tricarboxylic acid cycle, and cytochrome and toxin production and as a source of energy (Farnaud and Evans, 2003; Min et al., 2005). Reduction of environmental iron levels could eventually lead to less energy for pathogenic bacteria. Against gram-negative bacteria, lactoferrin targets the lipid A portion of the LPS layer, causing its release from the membrane, thus reducing bacterial survival (Wang et al., 2019). The antimicrobial mechanism against gram-positive bacteria is not well known but is thought to target teichoic acid in the bacterial cell wall; moreover, evidence indicates that the effect of lactoferrin may be even greater against some gram-positive bacteria than against gram-negative bacteria (Jahani et al., 2015).

Our results demonstrate that bovine lactoferrin does have a degree of antimicrobial properties against common dairy pathogens, specifically gram-negative bacteria, although at levels approximately 10 to 20 times higher than naturally found in bovine milk. The natural levels did not seem to have an inhibitory effect on either *S. enterica* or *E. coli* O157:H7 at 37°C or at ambient temperature (23°C). The MIC of lactoferrin was statistically determined using a mixed-effects model. This model was chosen to account for several random effects within the assay design. First, because the TSB and milk assays were repeated twice, the microtiter plate number from which each absorbance value originated was considered for any variation observed between the plates for each respective assay. Second, the row number on each plate was considered in the analyses because the wells were not randomized when setting up the assay. Last, the evaporation effect observed for each assay was taken into account when statistically analyzing these data. This partial loss of volume in multiple wells from evaporation during the 48-h incubation period, when graphed, resulted in curved lines for the controls, when ideally these values should remain constant.

The MIC of lactoferrin in TSB (3.516 and 7.031 mg/mL) increased to 14.063 and 112.5 mg/mL when performed in bovine milk. This effect could be attributed to cations in milk, such as calcium, that could slightly inhibit the effect of lactoferrin at the bacterial cell surface. Bennett et al. (1981) discovered that lactoferrin forms tetramers in the presence of calcium, which inhibits the ability of lactoferrin to bind to and damage the LPS layer. These results are comparable with similar food preservation studies. Chantaysakorn and Richter (2000) examined the effect of lactoferrin on *E. coli* and found that the addition of pepsin-degraded lactoferrin at 0.5 mg/mL to peptone-yeast glucose medium significantly inhibited the growth of *E. coli* but did not in carrot juice until the concentration was greater than 10 mg/mL. Brown et al. (2008) looked at the effect of lactoferrin in chitosan edible films against *E. coli* and *Lis. monocytogenes* and found that up to 2 mg/mL, lactoferrin alone did not significantly inhibit the growth of either bacterium until the protein was paired with nisin or EDTA. Ellison et al. (1988) found that the addition of these compounds enhanced the bactericidal activity of lactoferrin by acting as chelators to gather and bind the calcium attached to LPS and help release it from the outer membrane. Nisin also creates holes in the bacterial membrane to cause further damage to the bacterium. In a similar line of reasoning, the lactoperoxidase (**LP**) system has been well explored for biopreservative potential (World Health Organization, 2005), although it should be underscored that the use of natural agents such as lactoferrin or the LP system is not intended to ensure raw milk safety but rather to maintain initial quality. Ponce (2005) showed that LP system-treated raw milk held at 30 to 35°C inhibited the growth of a variety of pathogenic bacteria (a static effect); Wray and McClaren (1987) measured the bactericidal effects of the LP system in raw milk against *Salmonella*; and Parry-Hanson et al. (2009) published a similar LP system study focusing on cidal effects against *E. coli* O157:H7. Thus, the LP system represents a suitable and possible alternative to lactoferrin for biopreservative potential in raw milk.

The measurable differences we observed between *Salmonella* and *E. coli* O157:H7 when treated with lactoferrin might be attributed to the makeup of the O-side chains in the LPS of both bacteria. Hu et al. (2010) found that the O-antigens associated with the LPS vary from one bacterial species to another due to the different sugars, linkages, and arrangement possibilities, which may affect the efficacy of lactoferrin binding and damaging part of the LPS. For example, the O-antigens associated with LPS comprise 1 to 6 various sugar residues that can have slightly different charges, although the overall net charge is negative. Ammons and Copie (2013) postulated that the electrostatic differences may anchor the antigens too strongly to the outer membrane for lactoferrin to effectively cause release from the membrane. A second explanation for the measurable differences between *Salmonella* and *E. coli* could be phase and antigenic variation, or the ability to alternatively express certain structural genes and surface proteins. Both *S. enterica* and *E. coli* use phase variation to evade host immune defenses to prolong infection. *Salmonella enterica* uses phase variation for variable expression of flagellar genes as well as fimbriae to aid the bacteria in movement and adhesion in response to toxins or host defenses (van der Woude and Baumler, 2004). In addition, Broadbent et al. (2010) found that *E. coli* also uses phase variation for fimbriae as well as for lipo-oligosaccharide and outer membrane protein modifications. These changes in the structure of a bacterial cell increase pathogenicity and may create an additional obstacle for lactoferrin to attach to the membrane and cause a bactericidal effect.

Additionally, if environmental iron levels are low, some bacteria, such as those involved in biofilm formation or siderophore production, will express certain virulence genes to aid in their survival. However, it seems unlikely that *S. enterica* or *E. coli* O157:H7 formed biofilms in the microtiter plate wells because lactoferrin has been reported to prevent adhesion and biofilm formation. Ochoa et al. (2006) found that lactoferrin disrupts bacterial surface proteins and type III secretion systems via serine protease activity and binding of the lipid A portion of LPS to disrupt anchored virulence proteins. Another virulence factor that some bacteria, such as *S. enterica*, express under stress is siderophores to seek out and obtain ferric iron (Kaneshige et al., 2009). The low iron levels, however, do not drastically hinder the activity of lactoferrin against gram-negative pathogens because lactoferrin has both bacteriostatic and bactericidal effects. These are due to the 2 forms of lactoferrin: apo and holo. The holo form binds to the free iron, whereas the apo form binds to lipid A of the LPS outer membrane independently of iron levels to cause a bactericidal effect.

Because our MIC was 10- to 20-fold higher compared with the natural level in raw bovine milk, there are a few things to consider when discussing the potential of lactoferrin as a biopreservative measure. One is whether the increase in lactoferrin concentration would change the texture, aroma, or taste of raw milk. Shashikumar and Puranik (2011) reported that the addition of 20 ppm (~0.02 mg/mL) lactoferrin to paneer, a traditional Indian milk product, significantly changed the chewiness and hardness of the product after undergoing chemical, microbiological, and sensory analyses. However, the investigators did not inoculate paneer with pathogens but instead left the cheese at normal conditions to test lactoferrin against any type of microorganism, which may have a direct bearing on the lactoferrin concentration found to be effective. To determine whether our MIC would cause such consumer sensory changes to the milk, we would need to conduct sensory panels similar to those in the paneer study. A second aspect to consider when adding lactoferrin to milk is whether this would cause allergic reactions in consumers. Little research has been done to address bovine lactoferrin as an allergen. Negaoui et al. (2016) showed that bovine lactoferrin (2–10% wt/vol) produced a strong allergic reaction in mice and found that lactoferrin interacts with human IgE and might cross-react with other whey proteins.

The application of lactoferrin as a biopreservative in developing countries is still relative due to varied rates of urbanization in particular regions. Many families living in rural areas rely on milk production to improve their livelihoods, but areas with warmer climates year-round, such as sub-Saharan Africa or the Indian subcontinent, have a harder time producing milk (Knips, 2006; Moran and Chamberlain, 2017). The average annual temperature in these regions can range from 65 to 80°F, which can cause stress on the cows and lead to lower production yields. If fresh milk is not used by the farmer's family, it is sold to local vendors, who likely travel deep into the countryside on foot or by bicycle to transport the milk back to local markets. Once the milk is purchased, consumers usually heat the milk or ferment it to reduce the chance of illness or spoilage. Outside of rural areas, many countries import their milk in powder form or dairy farmers are contracted to work for larger companies or corporations (Davis et al., 2018).

The reality of using bovine lactoferrin as a potential biopreservative in dairy milk at a concentration of 3.516 mg/mL is practical but might be costly. In theory, lactoferrin should remain stable if added back into raw milk and should bind to the iron present due to the high affinity of binding (dissociation constant K_D_ = 10^−20^ *M*). Lactoferrin is a stable protein under a wide array of environmental conditions, especially when bound to iron. Saito et al. (1994) discovered that lactoferrin can withstand pasteurization temperature under acidic pH 2 to 5 and retain its physiochemical properties but can become easily denatured with heat above pH 6. However, there has been little research on the stability and activity of lactoferrin at refrigeration temperatures (~4°C). Shashikumar and Puranik (2011) tested the effectiveness of lactoferrin on paneer using refrigeration temperature in the design and found that lactoferrin increased the shelf life of paneer by 8 d compared with a control, indicating that lactoferrin seems to be stable and active around 4°C.

Overall, this study provides relevant information on bovine lactoferrin as an antimicrobial against 2 high-profile gram-negative pathogens, although it may not be applicable if used alone. Bovine lactoferrin had a more significant inhibitory effect on the growth of both *S. enterica* and *E. coli* O157:H7 in TSB than in milk. Future studies of lactoferrin could incorporate gram-positive dairy pathogens (*Bacillus cereus* or *Listeria monocytogenes*) to test the antimicrobial properties of lactoferrin on a broader scale and to more precisely examine whether lactoferrin would be appropriate as a value-added biopreservative agent. In addition to testing lactoferrin activity against selected bacterial pathogens, other dairy contaminants such as yeast (*Kluyveromyces, Candida, Cryptococcus*), protozoa (*Giardia* and *Cryptosporidium* spp.), and viruses (hepatitis A, norovirus, adenovirus) could be studied as well. An interesting alternative to investigate could be human lactoferrin instead of bovine lactoferrin. The 2 protein structures are quite similar, sharing 69% sequence homology. The main structural differences between human and bovine lactoferrin are the number of sugar chains, sialic acid content, and glycosylation sites (Shimazaki, 2000; Jiang et al., 2014; Karav et al., 2017). The difference in glycosylation could lead to changes in binding to bacterial membranes and activity of lactoferrin. The use of human lactoferrin may decrease the allergen potential compared with bovine lactoferrin and may be safer to use in higher concentrations. The application of such natural antimicrobials to dairy milk or dairy products could help alleviate the contamination potential in developing countries after processing and reduce the overall number of foodborne illnesses.
